# Distinct responses of wheat microbial communities in phyllosphere and rhizosphere to *Puccinia striiformis* infection

**DOI:** 10.3389/fmicb.2025.1639152

**Published:** 2025-10-07

**Authors:** Feng Gao, Liya Ma, Xiao Chen, Xin Xue, Junhong Hou, Junhong Dong, Weimin Shen, Chunling Yang, Yu Shi

**Affiliations:** ^1^Anyang Academy of Agriculture Sciences, Anyang, Henan, China; ^2^State Key Laboratory of Crop Stress Adaptation and Improvement, School of Life Sciences, Henan University, Kaifeng, Henan, China

**Keywords:** *Puccinia striiformis* (Pst), plant microbiome, phyllosphere, rhizosphere, disease resistance

## Abstract

In the plant ecosystem, microbiomes are of great significance in sustaining plant health, participating in multiple physiological activities like nutrient metabolism, stress resistance, and hormone regulation. However, the invasion of pathogens like *Puccinia striiformis* can disrupt the balance of the plant microbiome, significantly affecting plant growth, development, and metabolism. This study delved into the responses of wheat microbial communities in different niches, namely the phyllosphere and rhizosphere, to *P. striiformis* infection. The structure of the phyllosphere fungal community was predominantly affected by the wheat variety. The α–diversity of phyllosphere fungi increased with the enhancement of wheat resistance. In the rhizosphere, although the inoculation did not cause a notable alteration to the overall architecture of the bacterial and fungal communities, remarkable variations were detected in the relative proportion of certain microbial taxa across various resistant wheat varieties. The co-occurrence network of the rhizosphere underwent significant structural and functional reorganization, and the network became substantially more complex after inoculation. The study also uncovered the interaction among the microbial communities in the phyllosphere and rhizosphere, with highly resistant varieties showing a stronger ability to coordinate this interaction to optimize microbial community functions and enhance disease resistance. This research deepens the understanding of the wheat—*Puccinia striiformis*—microbial community interaction system and paves the way for further research on wheat disease prevention and control strategies.

## Highlights

- The α-diversity of phyllosphere fungi rises with increased wheat resistance.
- Invasion of pathogenic fungi into leaf zone impacts rhizosphere microbes.
- Moderately resistant wheat showed maximal inoculation-natural occurrence divergence.
- Inoculation of pathogens heightens rhizosphere microbial network complexity.

## 1 Introduction

In the life process of plants, the homeostasis of the plant microbiome is undoubtedly one of the core elements supporting plant's growth and metabolism (Santoyo, 2022; Teixeira et al., 2019). The plant microbiome is a microscopic ecosystem, containing a rich and diverse array of microbial taxa that are intricately linked to the plant host (Gao et al., 2021). These microorganisms are actively involved in the plant's nutritional metabolism processes. For instance, they could enable plants to take up essential nutrients like nitrogen and potassium through processes including nitrogen fixation and phosphorus solubilization, thereby enhancing the plant's utilization efficiency of nutrients in the soil (da Silva et al., 2023; Pang et al., 2024; Wei et al., 2024). The invasion of pathogens is like a sharp blade, mercilessly disrupting the balance of the plant microbiome. They can rapidly alter the microenvironment within plant tissues, especially the nutrients acquisition strategies and metabolites (Fatima and Senthil-Kumar, 2015; Pusztahelyi et al., 2015; Zaynab et al., 2018). These changes, in turn, lead to alterations in the structure and composition of plant-associated microbial communities (Batista et al., 2024). In response to pathogen invasion, plants tend to actively recruit more beneficial microorganisms, which are of vital importance for improving the plant's disease-resistance mechanisms (Trivedi et al., 2017; Wen et al., 2023). But some beneficial microorganisms that originally occupied a dominant position in the microbial community may be inhibited, and their growth, reproduction, and metabolic activities may be hindered (Hu et al., 2020). Meanwhile, certain pathogens or opportunistic pathogenic microorganisms may take advantage of the situation to multiply rapidly, causing an imbalance in the microbial community (Batista et al., 2024; Wei et al., 2018). This imbalance can undermine the plant's ability to resist stress, leaving the plant more exposed and at risk from various biotic factors like pests and pathogens, and abiotic stressors such as drought and extreme temperatures (Fang et al., 2025; Qiao et al., 2024; Trivedi et al., 2020). And in severe cases, it may even threaten the stability and balance of the entire ecosystem, triggering a chain reaction that affects the survival and reproductive capabilities of other organisms in the ecosystem (Hu et al., 2025). Thus, it is necessary to understand the responses of the plant microbiome when its balance is disrupted by pathogen invasion.

In the ecosystem of wheat, an important food crop, there are extremely complex and delicate connections and interactions between the rhizosphere and phyllosphere microbial communities (Aziz et al., 2021). The microbial community in the phyllosphere inhabits the surface of wheat leaves and the surrounding microenvironment. Its composition and function are shaped by multiple elements including the atmospheric environment, the physical and chemical characteristics of the leaf surface, and the physiological state of the plant (Liu et al., 2020; Xu et al., 2022). When the phyllosphere microbial community changes, it is highly probable that such alterations will be conveyed to the rhizosphere microbial community via a variety of routes (Gong and Xin, 2021). On the one hand, the above-ground and below-ground parts of plants conduct material transport and signal transmission through the vascular bundle system (Chang et al., 2022). Changes in the phyllosphere microbial community may trigger the plant to generate systemic signals, which are transmitted to the roots through the vascular bundle and then affect the function of the rhizosphere microbial community (Li et al., 2023; Philippot et al., 2013). On the other hand, the microorganisms on the leaf surface may migrate to the rhizosphere environment through rainwater erosion, air movement, or insect transport, directly participating in the processes of assembly and succession of the rhizosphere microbial community (Gong and Xin, 2021; Sohrabi et al., 2023). Consequently, the substances secreted by plant roots, such as phenolic compounds, organic acids, and amino acids, are also affected by alterations in the phyllosphere microbial community, indirectly changing the living habitat and community dynamics of the rhizosphere microorganisms (Feng et al., 2024; Vives-Peris et al., 2020; Yuan et al., 2018).

The rhizosphere, as a critical area for wheat to obtain nutrients and water, holds an essential and irreplaceable position in the growth of wheat (Affortit et al., 2024). Microorganisms in the rhizosphere can degrade soil organic matter and convert it into inorganic nutrients highly accessible to plants, such as converting complex organic nitrogen compounds into ammonium nitrogen and nitrate nitrogen to promote plant nitrogen absorption and utilization (Fang et al., 2025; Jiang et al., 2024; Xu et al., 2025; Zheng et al., 2022). Besides, some rhizosphere microorganisms can build symbiotic relationships with plant roots (Luo et al., 2025; Mommer et al., 2016). This augmentation enables plants to more effectively uptake water and nutrients. It promotes the growth rate of wheat and fortifies its stress - tolerance capabilities (van der Heijden et al., 2015). Furthermore, the rhizosphere microbial community is capable of generating substances including antibiotics and siderophores to suppress the growth and proliferation of pathogens, protecting the wheat roots from disease attacks and playing a vital part in maintaining wheat health and promoting growth and development (Gu et al., 2020; Hartmann and Six, 2023; Wang N. Q. et al., 2024).

Given the huge threat posed by wheat stripe rust to wheat production worldwide (Zeng et al., 2022; Zhao and Kang, 2023), in-depth exploration of the response mechanisms of wheat microbial communities in different niches after *Puccinia striiformis* infection has become a focal point of research in the fields of plant pathology and microbial ecology. Wheat stripe rust, caused by *Puccinia striiformis*, is a globally destructive airborne fungal disease (Schwessinger, 2017; Zhao and Kang, 2023). Under suitable climatic conditions, it can break out and spread over a large area in a short time, resulting in a sharp reduction in wheat yield and bringing heavy economic losses to agricultural production (Schwessinger, 2017). Therefore, it is of great urgency to address the following crucial scientific queries: How do the rhizosphere and phyllosphere microbial communities of different resistant wheat varieties respond uniquely when facing *Puccinia striiformis* infection? During the process of *Puccinia striiformis* infection, what specific shifts will occur in the operational aspects of wheat microbial communities in diverse niches? Through in-depth research on these questions, it is expected to uncover the intricate interaction mechanisms between wheat and *Puccinia striiformis*, provide a firm theoretical groundwork for developing new wheat disease resistance strategies based on microbial community regulation, and also provide important technical support and practical guidance for ensuring the safe production and sustainable development of wheat worldwide.

## 2 Materials and methods

### 2.1 Experimental site

This experiment was conducted on the farm (114.357 °E, 36.196 °N) of the Wheat Research Institute, Anyang Academy of Agricultural Sciences, Henan Province. Three wheat varieties were planted: the highly resistant variety to stripe rust, Anmai 1350 (AM1350, Approval No. Guoshenmai 20210136), the medium-resistant variety Anmai 11 (AM11, Approval No. Guoshenmai 20220102), and the susceptible variety Mingxian 169 (MX). For each variety, a control group and an inoculation group were established, each with 6 replicate quadrats. The quadrats, sized 4.5 m × 1.5 m, were organized according to a randomized block design. This setup allowed for a comprehensive investigation of the impact of wheat variety and *Puccinia striiformis* inoculation on the microbial communities in different experimental conditions.

### 2.2 Inoculation method

The stripe rust fungus used in the experiment was mixed culture of fungal isolates provided by the Plant Protection Research Institute of Gansu Academy of Agricultural Sciences, mainly composed of Guinong pathogenic groups (52.4%) including stripe 34 (dominant species, 40%), stripe 32 (15%), stripe 33, Gui22-14 and water source types, etc. On March 21, 2023, the inoculation group of wheat was inoculated with *Puccinia striiformis* using the powder - shaking method (Liang et al., 2018). At this stage, the plants were approximately 5 months old and at the early jointing stage, a period when wheat is particularly susceptible to *Puccinia striiformis* infection (Zeng et al., 2022; Zhao and Kang, 2023). The well - cultured fungal spores were filled into clean plastic bottles and sealed for later use. The bottle mouth was cut into a cross - shaped shape with a knife to facilitate the spraying of the spores. Each quadrat had 6 rows of wheat planted. In the afternoon of the inoculation day, the middle two rows of wheat leaves in each quadrat were wetted with water. Then, the plastic bottle containing the spores was held with the bottle mouth tilted downward, and the bottle body was squeezed to spray the spores onto the wet leaves. Immediately after that, a plastic film was laid to cover the quadrat tightly, and the edges were sealed with soil to maintain moisture overnight. After that, the plastic film was removed the next morning.

### 2.3 Sampling

Distinct disease symptoms appeared on April 12, and sampling was conducted after the large-scale onset of wheat disease on April 19. At this stage, the plants were approximately 6 months old and at the heading stage. From each treatment of each variety, three quadrats were randomly selected to collect rhizosphere soil and leaf samples. When sampling rhizosphere soil, five seedlings were collected from three sampling points within each quadrat and then combined to form a single sample. Before sampling, sampling tools were thoroughly cleaned first and then rubbed with the local soil near the sampling site to minimize external interference. All operations were performed with gloves to avoid contamination. The whole plant was extracted as completely as could be managed. Then the loose soil was removed. And the soil adhering to the roots within a 1-mm radius was gently brushed off and gathered as the rhizosphere sample, which was immediately placed in a self-sealing bag. Leaf samples were collected from the upper-middle part of the main stem of the wheat plant.

### 2.4 Sequencing and data analysis

The amplified products underwent sequencing through high-throughput sequencing methods on the Illumina NovaSeq platform provided by MAGIGENE Company (http://www.magigene.com/). The raw sequencing data were pre-processed using QIIME2. The paired-end sequences were combined and the primer sequences were eliminated through the cutadapt plugin. By clustering the raw sequencing data with 100% identity using DADA2, an Amplicon Sequence Variant (ASV) table was generated. For bacterial phylotype identification, the Silva138 release database was utilized, while the UNITE database was employed to assign fungal taxonomy. The sequencing depth per sample and rarefaction thresholds are as follows: for phyllosphere fungal samples, the sequence counts ranged from 64,232 to 176,121, with a rarefaction threshold of 64,232; for rhizosphere bacterial samples, sequence counts ranged from 80,745 to 167,117, with a rarefaction threshold of 80,745; for rhizosphere fungal samples, sequence counts ranged from 57,510 to 111,487, with a rarefaction threshold of 57,510. These thresholds were used to ensure comparability across samples. QIIME2 was used to compute various diversity indices of the microbial communities, including Shannon Entropy and Pielou Evenness (Pielou, 1966; Shannon, 1948). The results presented are aggregated from all biological replicates (3 per treatment group), with alpha diversity metrics averaged and relative taxonomic abundances averaged across replicates to depict community trends, and significant differences between groups were determined using non parametric and Tukey tests, respectively. To assess the β–diversity of communities, the Bray-Curtis and Jaccard distances were calculated with the vegan package. The Kruskal-Wallis H test in SPSS software was applied to analyze the differences in diversity among samples. Additionally, Principal Coordinate Analysis (PCoA) was carried out on the samples based on Bray-Curtis distances using the vegan package to visualize their distribution patterns (Oksanen et al., 2019). The common and unique ASVs of phyllosphere fungi in wheat with different resistances between the inoculation treatment and the control treatment were analyzed through the Venn analyse. Using Weighted Gene Co-expression Network Analysis (WGCNA) to construct co-occurrence network analysis, two networks were established for the inoculation treatment and the control treatment respectively, and three networks were constructed for three wheat varieties. Each network was established by rhizosphere bacteria and fungi. In each network, only the ASVs present in more than 40% of bacterial samples and 20% of fungal samples were retained.

## 3 Result

### 3.1 Phyllosphere microbial diversity and abundance responses to wheat variety and inoculation

**Figure 1:**
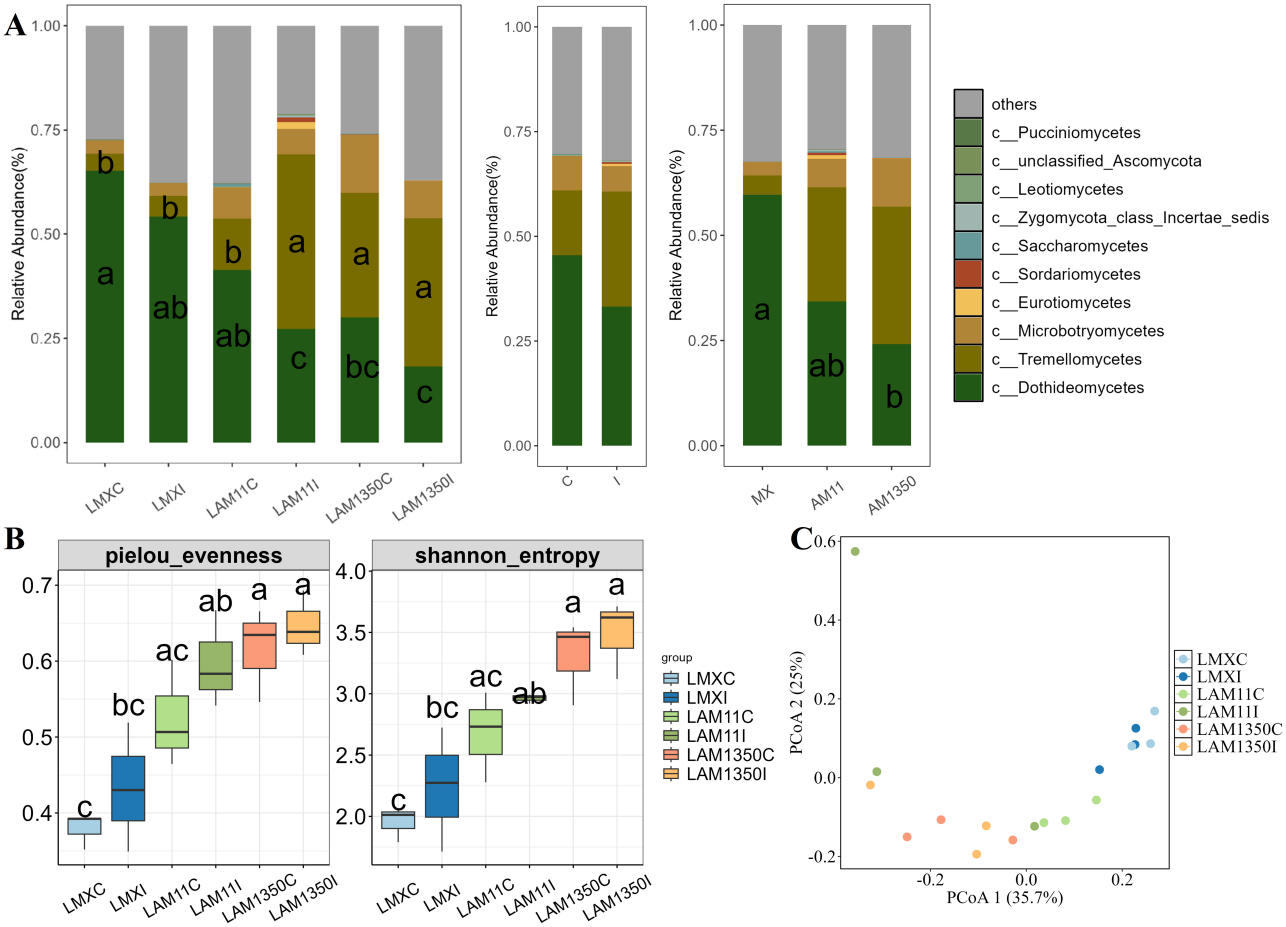
Relative abundance of the dominant fungal class **(A)**, α diversity **(B)** and principal coordinate analysis (PCoA), **(C)** across all samples of wheat phyllosphere. C, The control treatment; I, The inoculation treatment; LMXC, Leaf samples of Mingxian wheat in the control treatment; LMXI, Leaf samples of Mingxian wheat in the inoculation treatment; LAM11C, Leaf samples of Anmai 11 wheat in the control treatment; LAM11I, Leaf samples of Anmai 11 wheat in the inoculation treatment; LAM1350C, Leaf samples of Anmai 1350 wheat in the control treatment; LAM1350, Leaf samples of Anmai 1350 wheat in the inoculation treatment. The lowercase letters in the figures indicate significant differences between groups.

The relative abundance of Dothideomycetes in the phyllosphere changed remarkably (Figure 1A). In the highly susceptible variety Mingxian 169, its relative abundance could reach 0.6, whereas in the highly resistant variety Anmai 1350, it was only 0.23. Moreover, its relative abundance decreased in accordance with the enhancement of wheat resistance and the process from natural occurrence to artificial inoculation, which had a profound effect on the ecological functions and balance of the phyllosphere microbial community. Besides, the class Tremellomycetes exhibited contrasting abundance patterns: its relative abundance was obviously higher in the highly resistant AM1350 than the other varieties (Figure 1A). And inoculation with *Puccinia striiformis* triggered a significant increase in Tremellomycetes abundance in the moderately resistant AM11 (*p* < 0.05, Figure 1A). The α-diversity of phyllosphere fungi exhibited a distinct and regular pattern of change (Figure 1B). Taking the Shannon index as an example, it reached 3.5 in the highly resistant variety Anmai 1350 after inoculation, while it was only 2.0 in the highly susceptible variety Mingxian 169. Overall, it increased with the enhancement of wheat resistance and the progression from natural occurrence to artificial inoculation (Figure 1 and Supplementary Figure S1).

**Figure 2:**
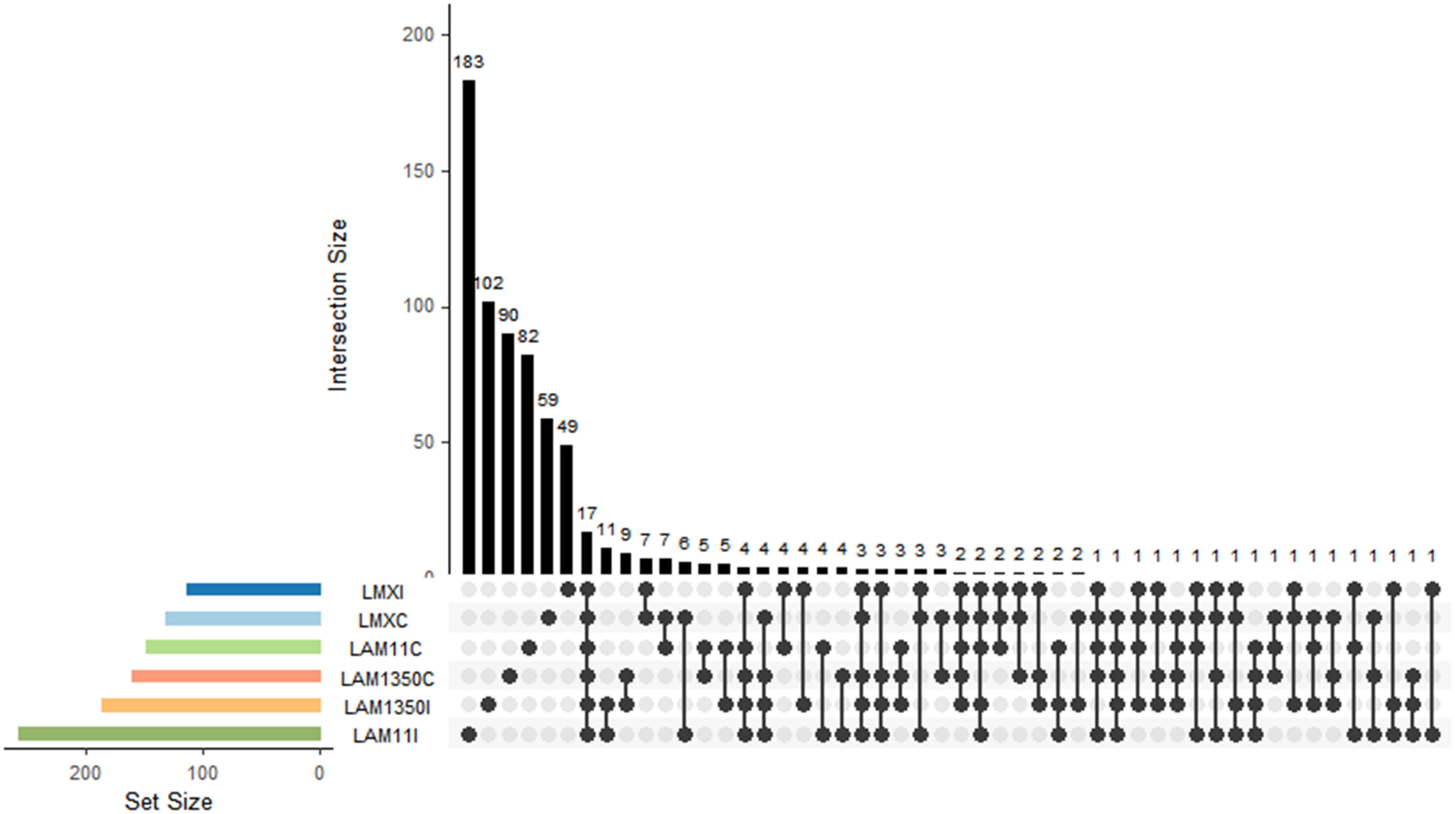
Intersection of fungal taxa among different treatments and wheat varieties. The bar chart shows the intersection size of fungal taxa combinations across groups, and the venn-like dot diagram below illustrates the intersection relationships of fungal taxa among groups. C, The control treatment; I, The inoculation treatment; MX, Samples of Mingxian wheat; AM11, Samples of Anmai 11 wheat; AM1350, Samples of Anmai 1350 wheat.

Permanova analysis results demonstrated that the influence of wheat varieties on the phyllosphere fungal community structure was significant. In the analysis based on Jaccard distance (*R*^2^ = 0.331, *P* = 0.002) (Supplementary Figure S1 and Table 1), it distinctly demonstrated that the variety factor was the primary determinant in forming the structure of the phyllosphere fungal community, whereas the inoculation treatment had an insignificant influence on the structure of the phyllosphere fungal community (Supplementary Figure S2). In addition, it was shown that wheat varieties with different resistances respond differently to inoculated pathogens. Among them, the moderately resistant variety AM11 shows the most significant changes (Figure 2). The number of fungi unique to the inoculation treatment of AM11 reaches 183, which is much higher than that of other varieties under inoculation treatment. Moreover, the number of fungi common to the control and inoculation treatments is relatively small, only 4, highlighting that the structure of the leaf microbial community of AM11 has undergone significant changes after inoculation with pathogens. In contrast, the variations in the number of fungi of varieties MX and AM1350 under corresponding treatments are relatively smaller compared to that of variety AM11. Specifically, the fungal species Aspergillus (belonging to the Ascomycota family) was only detected in the inoculated wheat varieties, and was found in all three wheat varieties after inoculation.

**Table 1:** PERMANOVA analysis of the effect of variety, treatment and variety × treatment on the dissimilarity of phyllosphere fungi and rhizosphere bacterial and fungal communities.

**Factor**	**Phyllosphere fungi**		**Rhizosphere bacteria**		**Rhizosphere fungi**	
	***R** ^2^*	***P***	***R** ^2^*	***P***	***R** ^2^*	***P***
Variety	**0.331**	**0.002** ^**^	0.130	0.201	0.135	0.141
Treatment	0.055	0.219	0.060	0.377	0.063	0.274
Variety × treatment	0.096	0.325	0.111	0.621	0.121	0.324

^**^Bold mean significant difference.

The relative abundances of diverse fungal functional groups vary significantly across samples (Supplementary Figure S2). In LAM11I, functional groups such as Wood Saprotroph, Undefined Symbiotroph, and Soil Saprotroph show relatively higher proportions compared to other samples. And the Plant Saprotroph group exhibits distinct patterns: its relative abundance in LAM11I and LAM1350I is higher than in LMXC and LMXI. Moreover, the abundance of some groups (e.g., Endosymbiont) changes with the inoculation state. Groups such as Animal Symbiotroph maintain a relatively stable, albeit low, abundance across most samples, while others like Epiphyte and Endophyte display sample-specific fluctuations that could be linked to sample characteristics and potential environmental or experimental triggers.

### 3.2 Rhizosphere microbial community in response to wheat variety and inoculation

#### 3.2.1 Diverse responses of rhizosphere microbial diversity and species abundance among different wheat varieties to inoculation

**Figure 3:**
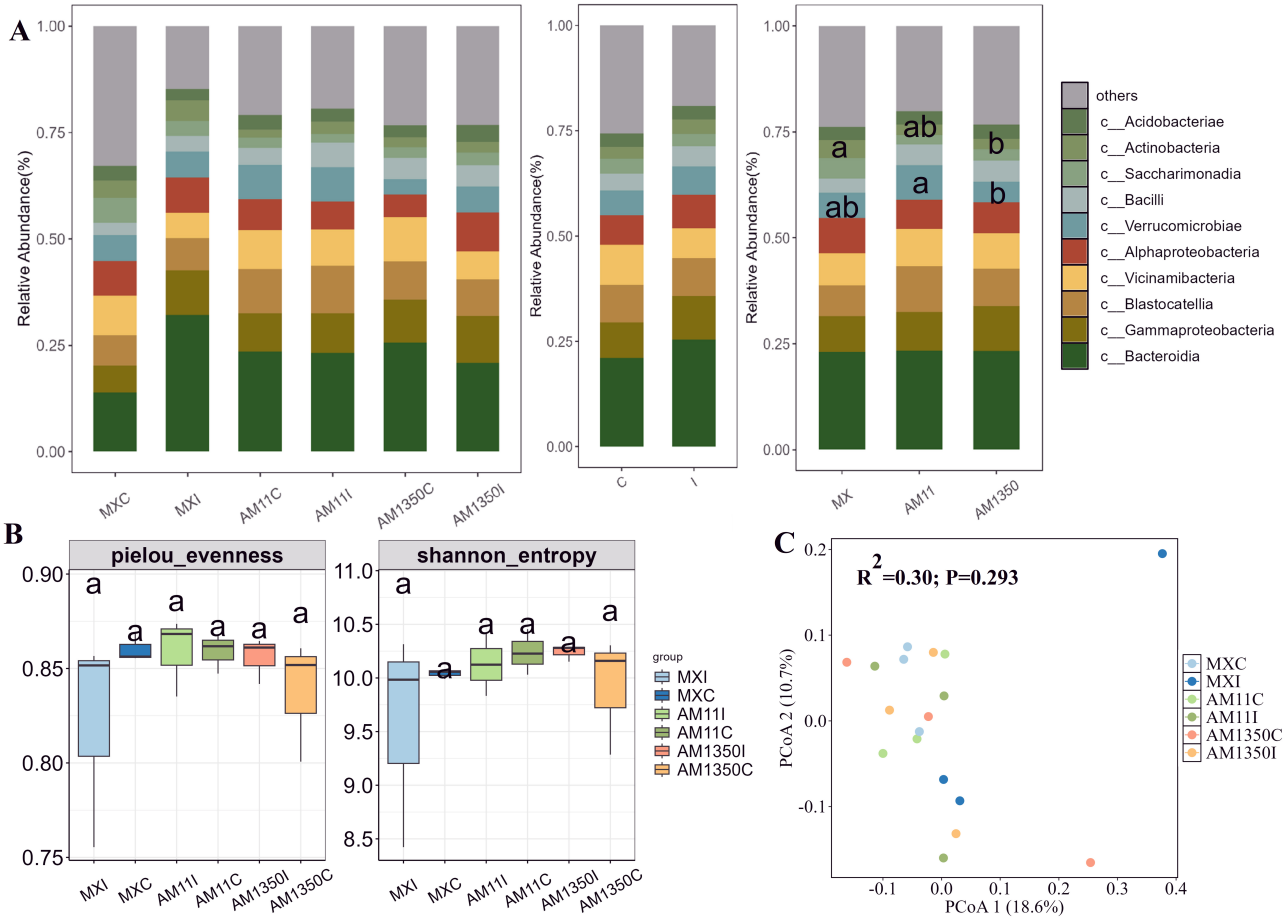
Relative abundance of the dominant bacterial class **(A)**, α diversity **(B)** and principal coordinate analysis (PCoA), **(C)** across all samples of wheat rhizosphere. C, The control treatment; I, The inoculation treatment; MXC, Root samples of Mingxian wheat in the control treatment; MXI, Root samples of Mingxian wheat in the inoculation treatment; AM11C, Root samples of Anmai 11 wheat in the control treatment; AM11I, Root samples of Anmai 11 wheat in the inoculation treatment; AM1350C, Root samples of Anmai 1350 wheat in the control treatment; AM1350I, Root samples of Anmai 1350 wheat in the inoculation treatment. The lowercase letters in the figures indicate significant differences between groups.

**Figure 4:**
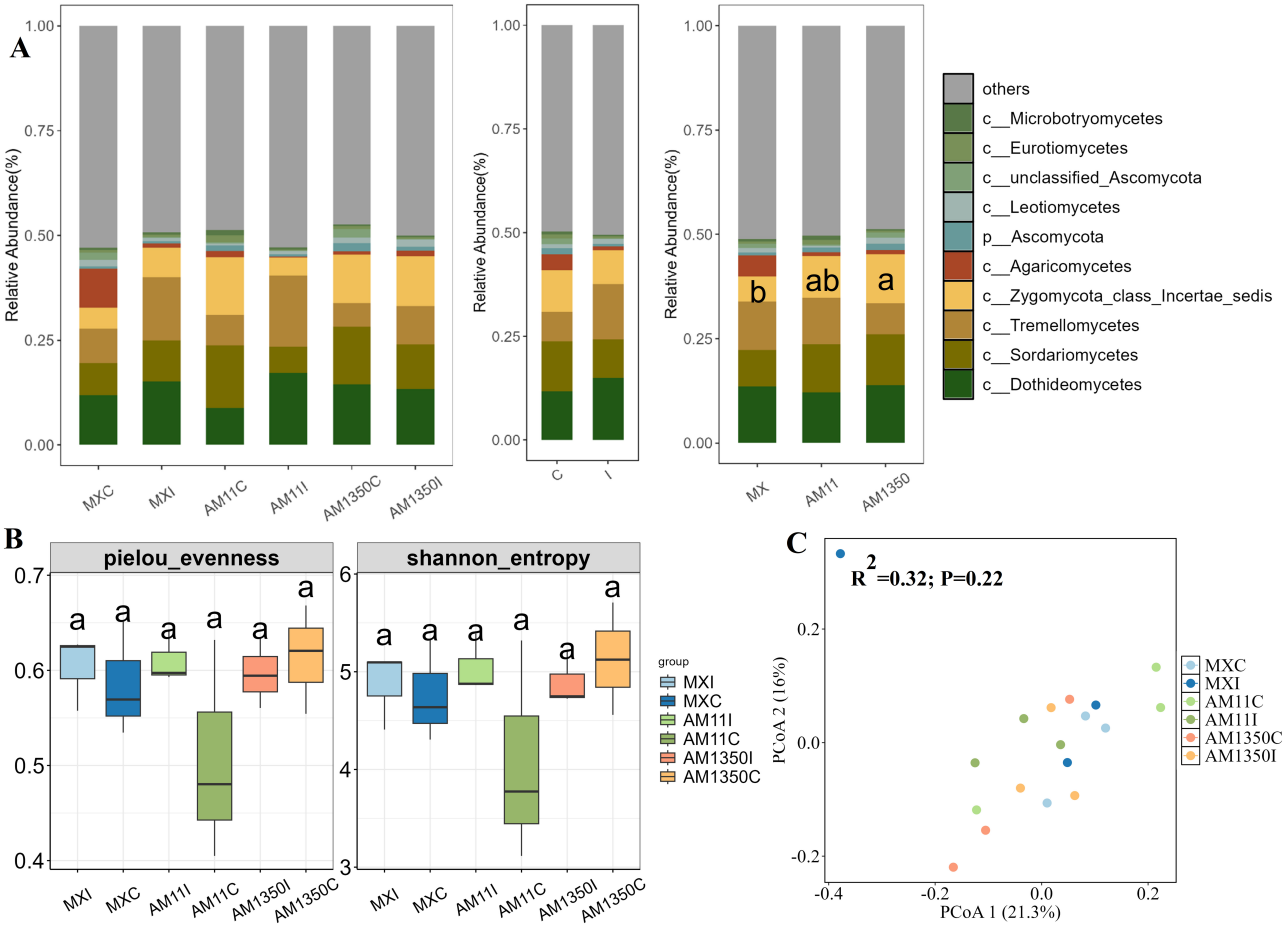
Relative abundance of the dominant fungal class **(A)**, α diversity **(B)** and principal coordinate analysis (PCoA), **(C)** across all samples of wheat rhizosphere. C, The control treatment; I, The inoculation treatment; MXC, Root samples of Mingxian wheat in the control treatment; MXI, Root samples of Mingxian wheat in the inoculation treatment; AM11C, Root samples of Anmai 11 wheat in the control treatment; AM11I, Root samples of Anmai 11 wheat in the inoculation treatment; AM1350C, Root samples of Anmai 1350 wheat in the control treatment; AM1350I, Root samples of Anmai 1350 wheat in the inoculation treatment. The lowercase letters in the figures indicate significant differences between groups.

After inoculation, the relative abundance of the genus Bacteroides in the rhizosphere of Mingxian increased significantly from 0.13 to 0.28 while decreased in AM1350 (Figure 3A and Supplementary Figure S3). In the meantime, the inoculation resulted in a tendency to increase in the relative abundances of Tremellomycetes and Dothideomycetes within the rhizosphere (Figure 4A and Supplementary Figure S4), and these changes further altered the functionality of the rhizosphere microbial community. In the bacterial community, the AM1350 group exhibited a notably greater abundance of bacteria with metabolic functions such as xenobiotics biodegradation and drug metabolism compared to other groups. The AM11 group only showed prominence of bacterial groups associated with the metabolism of cofactors and Biotin. In the fungal community, Symbiotroph was much more abundant in the AM1350 group than in the other groups, reflecting the uniqueness of this taxon in the AM1350 group.

Although no significant differences in rhizosphere bacterial α-diversity were detected among groups, inoculation triggered divergent trends in Pielou evenness indices: the highly susceptible Mingxian 169 trended to increase, whereas Anmai 1350 and Anmai 11 trended to decrease (Figure 3B). Similar trend was found in Shannon index. As for the α-diversity of rhizosphere fungi, the difference in the pielou evenness index between Anmai 1350 and Anmai 11 reached 0.15. Additionally, the medium-resistant variety Anmai 11 showed a relatively large difference between inoculated and non-inoculated conditions, fully reflecting that the responses of the rhizosphere bacterial and fungal communities of different resistant varieties to *Puccinia striiformis* infection were different (Figures 3B, C, and 4B, C).

#### 3.2.2 Inoculation-induced changes in construction and interaction of rhizosphere microbial communities

**Figure 5:**
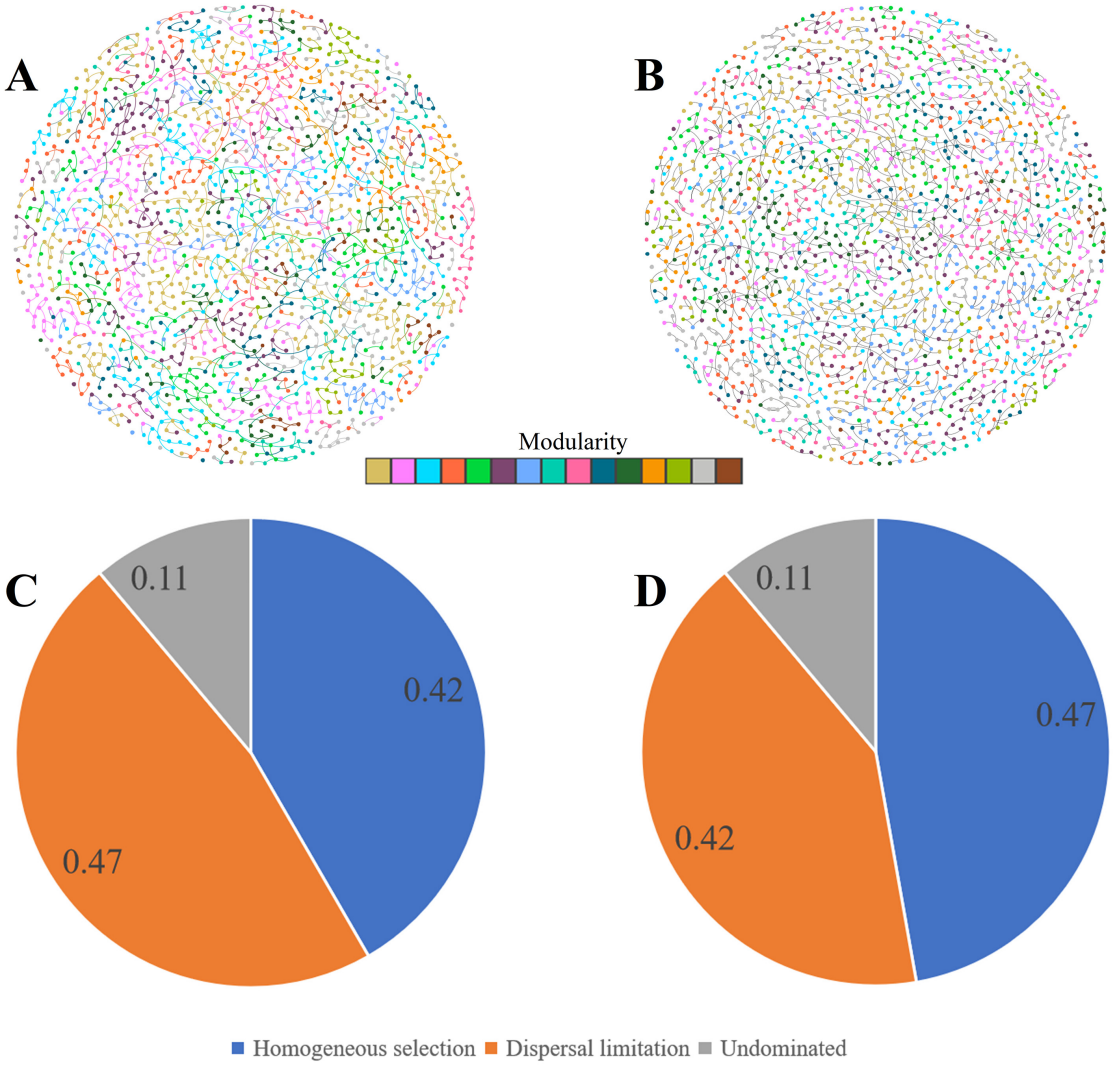
Rhizosphere microbial co-occurrence networks and bacterial community assembly mechanisms under control and inoculation conditions. **(A, B)** Bacterial-fungal crossover co-occurrence networks in control **(A)** and *Puccinia striiformis*-inoculated **(B)** rhizosphere using WGCNA with strict thresholds (Spearman's *r* > 0.6, *p* < 0.01) for correlations between ASVs, visualized via Cytoscape. Nodes represent ASVs, edges denote significant correlations (width ∞ r), and colors indicate modules. **(C, D)** Bacterial community assembly processes inferred via Stegen's null model algorithm in control **(C)** and inoculated group **(D)**.

The rhizosphere microbial community of both the inoculated and non-inoculated groups were mainly dominated by stochastic processes (Figures 5A, B). However, the proportion of deterministic processes in the control group reached 47%, which was slightly higher than that in the inoculated group. This indicated that *Puccinia striiformis* infection changed the randomness of rhizosphere microbial community assembly to a certain extent, but the rhizosphere microbial community still retained a certain degree of stability and self-regulatory ability.

In comparison to the group without inoculation, the inoculated group demonstrated modifications in several topological properties of the network (Figure 5 and Supplementary Table S1). The global clustering coefficient, average clustering coefficient, eigenvector centralization, betweenness centralization, and degree centralization all increased slightly. And the complexity also increased from 12.69 to 13.40. However, the edge density remained the same between the two groups, and the modularity was also identical. In addition, the number of clusters in the uninoculated group was 4, while that in the inoculated group was only 1. Notably, the network stability in the inoculated group was inferior to that in the non-inoculated group. These results indicate that inoculation with *Puccinia striiformis* has changed the structure and connection characteristics of the rhizosphere bacterial-fungal cross-domain network to a certain extent, but at the same time, it has reduced the network stability (Supplementary Figure S5).

Among the three wheat varieties with different resistance levels, the highly resistant variety AM1350 showed uniqueness in several indicators (Supplementary Table S1). The number of clusters of AM1350 was the highest, reaching 16, compared with 15 in AM11 and 14 in MX. In terms of centrality-related indicators, the degree centralization of AM1350 was 0.06, and the betweenness centralization was 0.02, both of which were higher than those of the other two varieties; its global clustering coefficient was 0.55, and the average clustering coefficient was 0.54, also at a relatively high level; in terms of complexity indicators, AM1350 far exceeded AM11 (14.12) and MX (14.32) with a value of 18.70. Although in terms of modularity, both AM11 and MX had a value of 0.69, slightly higher than 0.63 of AM1350, overall, the rhizosphere bacterial-fungal cross-domain network structure of the highly resistant variety AM1350 was more complex, and it had obvious advantages in the influence of key nodes in the network and the overall complexity of the network.

## 4 Discussion

This study focuses on the microbial communities in different niches of wheat infected by *Puccinia striiformis*. The results reveal significant changes at multiple levels, and these findings are closely intertwined with the theories expounded in the preface, providing crucial clues for a more profound comprehension of the wheat—*Puccinia striiformis*—microbial community interaction system.

From the perspective of plant microbiome homeostasis, the infection of *Puccinia striiformis* disrupts the initial equilibrium of the microbial community, which aligns with the well-established understanding in plant pathology research (Lv et al., 2023). In the phyllosphere, although the fungal community structure is mainly dominated by the variety, there are still subtle changes after inoculation. This suggests that *Puccinia striiformis* interferes with the stable state of the phyllosphere microbiome to some extent, as has already been discovered in previous research (Chen Y. et al., 2022; Zhang et al., 2023). Notably, phyllosphere bacterial community analysis was unsuccessful due to low ASV richness, and microbial network construction was precluded by substantial inter-variety community variations and limited sample sizes. Consequently, co-occurrence network analysis of the phyllosphere microbiome was not conducted. The increase in fungal diversity in the phyllosphere of highly resistant varieties may be the result of the activation of their own defense mechanisms. During the process of resisting *Puccinia striiformis*, plants may secrete specific substances to attract beneficial fungi and reshape the phyllosphere microbial community structure to enhance the ability of resistance. For example, plants secrete specific substances, such as siderophores, enzymes, and signaling molecules, upon pathogen invasion, which can specifically recruit new microbes like Pantoea and Methylobacterium, and enrich native beneficial bacteria such as Sphingomonas to suppress pathogens through mechanisms including iron competition and cell wall degradation (Li et al., 2022). Additionally, Jia et al. (2023) successfully isolated a biocontrol streptomycete, which showed significant efficacy in inhibiting the invasion of *Puccinia striiformis*. This mechanism aligns with the “cry for help” strategy, whereby plants under biotic stress actively release signaling molecules to recruit beneficial microorganisms, observed in rhizosphere and other plant-microbe interaction studies, providing a theoretical basis for utilizing phyllosphere microbes in plant disease control (Mendes et al., 2013). A recent study by Wang's research group has provided compelling evidence for this hypothesis. Their findings indicate that when rice resists *Puccinia striiformis*, it secretes 2,4-di-tert-butylphenol (2,4-DTBP) from Aspergillus cvjetkovicii to attract beneficial fungi, reshape the phyllosphere microbial community, enhance ecological niche stability, and resist pathogen invasion, reducing field disease indices by 51-70% (Fan et al., 2024). Besides, the observed shifts in specific phyllosphere fungal taxa—correlated with wheat resistance levels and *Puccinia striiformis* inoculation—align with well-documented roles of these groups in plant defense. For instance, the reduced abundance of Dothideomycetes in resistant varieties is noteworthy, as this class includes known plant pathogens such as Alternaria and Septoria spp. (Stergiopoulos et al., 2013). Their lower presence in resistant genotypes may reflect diminished pathogen colonization potential or reduced synergism with rust fungi, reinforcing a link between Dothideomycetes dynamics and host resistance. Similarly, the class Tremellomycetes fungi, which exhibit significant differences in leaf tolerance among different resistant wheat varieties, may play an important role in helping wheat resist pathogens. Studies have found that yeasts of the genus Vishniacozyma in the grain of Canadian prairie wheat were significantly associated with wheat resistance to Tilletia and Fusarium (Vujanovic, 2021). These yeasts can inhibit pathogen colonization by occupying ecological niches, and may also synergize with endophytes like Penicillium and parasitic fungi such as Sphaerodes to regulate the microbial community structure for pathogen suppression (Li Y. et al., 2025; Vujanovic, 2021). Such findings strengthen the potential role of Tremellomycetes in wheat disease resistance, supporting the idea that phyllosphere microorganisms hold substantial promise for enhancing plant defense. Besides, functional inference via databases such as FUNGuild preliminarily suggests that the potential functions of enriched taxa (e.g., Endosymbiont) are associated with disease resistance. However, these inferences remain indirect. Future studies will detect the expression of defense genes via qPCR and quantify salicylic acid, jasmonic acid, and other hormones using LC-MS/MS to directly validate the associations between microbial community shifts and plant immune pathways, clarifying their causal relationships and regulatory mechanisms, thereby laying a foundation for unraveling microbial-driven wheat resistance to stripe rust (Nakagami et al., 2024; Pan et al., 2016; Wang F. Y. et al., 2024).

In the rhizosphere, although the structure of the bacterial and fungal communities is not markedly influenced by inoculation, the changes in some microbial taxa cannot be ignored. For example, upon infection of wheat by the wheat yellow mosaic virus (WYMV), the rhizospheres of healthy plants are abundant in beneficial microorganisms such as Sphingomonas. In contrast, the rhizospheres of diseased plants harbor a higher proportion of potential pathogens, which reveals a change in microbial species composition in response to the disease (Wang F. Y. et al., 2024). Likewise, when wheat is infected by Rhizoctonia solani AG8, the quantities of genera with potential antagonistic capabilities, such as Chitinophaga, Pseudomonas, Chryseobacterium, and Flavobacterium, rise (Yin et al., 2021). This represents an obvious alteration in microbial taxa related to the plant's reaction to the pathogen. When considering wheat cultivars that exhibit varying degrees of resistance to Fusarium head blight, the rhizosphere soil of resistant wheat cultivars contains a reduced abundance of Fusarium graminearum and a greater relative abundance of beneficial genera like Arthrobacter and Pseudomonas, demonstrating that plant resistance levels can influence rhizosphere microbial taxa (Li et al., 2020). In the present study, the elevation in the genus Bacteroides in Mingxian may be a self-regulatory mechanism employed by the rhizosphere microbial community to cope with the stress imposed by *Puccinia striiformis*. This further provides evidence that under the stress of pathogens such as *Puccinia striiformis*, plants tend to recruit more bacteria from the phylum Bacteroidetes to assist in nutrient acquisition and stress resistance. Studies have demonstrated that upon the invasion of pathogens into plants, these plants are capable of secreting particular substances into the rhizosphere, thereby attracting beneficial microorganisms (Trivedi et al., 2017). In this process, Bacteroidetes bacteria are among the recruited ones. For example, they may favor specialization in organic phosphorus mineralisation, making them more accessible to plants, which is crucial for plants to enhance their stress resistance during pathogen attacks (Lidbury et al., 2021). This further validates the crucial role of rhizosphere microorganisms in maintaining plant health. In future studies, functional validation of key microbial taxa identified herein is needed. This includes isolating pure cultures via selective media, conducting *in vitro* antagonism assays, and performing pot inoculation experiments (Köhl et al., 2019; Shi et al., 2024; Yu et al., 2024). Such work will clarify their roles in disease suppression and plant health, laying the groundwork for developing targeted microbial inoculants. Moreover, studies have found that after pathogen infection, the phylum Bacteroidetes assumes an extremely important role in the microbial co-occurrence network, helping maintain the relative homeostasis of the rhizosphere microbiome (Carrión et al., 2019). However, network dynamics are multifaceted, and network complexity and stability are not necessarily correlated. In this study, inoculation with *Puccinia striiformis* increased network complexity while reducing stability, aligning with findings in alpine slope ecosystem communities where microbial shifts toward more resource-use-efficient K-strategies increase network complexity yet undermine system robustness (Wang M. et al., 2024). And the network characteristics of the highly resistant variety AM1350 provide a unique perspective. Despite its lower modularity, its high complexity and the largest number of clusters imply alternative stability mechanisms. Such patterns may reflect functional redundancy within clusters, where multiple taxa perform overlapping roles to buffer against disturbance (Chen H. et al., 2022). This aligns with recent findings that microbial networks in resistant plants often evolve context-dependent stability strategies, prioritizing functional complementarity over strict modular separation (Trivedi et al., 2020). The genotype-dependent microbial responses, such as the distinct network traits of the highly resistant variety AM1350, provide a basis for developing microbiome-based breeding markers or tailored microbial inoculants. Such genotype-specific strategies hold promise for enhancing stripe rust resistance by leveraging unique host-microbiome associations across different wheat varieties.

Although changes in the phyllosphere microorganisms do not directly reshape the structure of the rhizosphere microbial community, fluctuations in the relative abundances of certain microbial taxa and alterations in network characteristics strongly suggest the existence of latent channels for signal transmission and material exchange between these two niches. Upon infection by pathogen, plants experience significant hormonal and metabolic shifts (Jin et al., 2024; Xu et al., 2022). For example, the synthesis of salicylic acid (SA) and jasmonic acid (JA) frequently experiences a significant increase (Eichmann et al., 2021). Salicylic acid is recognized for its pivotal function in the plant's defense mechanisms, and it can be triggered by the onslaught of pathogens (Li J. et al., 2025; Zhang et al., 2024). As demonstrated in Jia et al.'s study on Streptomyces tauricus XF, which inhibits *Puccinia striiformis*, the infection leads to the induction of SA-related genes such as PR1, PR2, and PAL in wheat leaves (Jia et al., 2023). This increase in SA not only targets the pathogen but also has an impact on the associated microbial communities (Eichmann et al., 2021; Jia et al., 2025). The changes in the phyllosphere can then send signals to the rhizosphere through the plant's vascular system or via the release of volatile compounds (Chang et al., 2022; Philippot et al., 2013). Moreover, the infection triggers the plant to secrete specific metabolites. In the research on Panax notoginseng infected by foliar pathogens, it was discovered that infection on the leaves promoted the release of both short-and long-chain organic acids, sugars, and amino acids from the roots (Luo et al., 2022). Also, as plants are repeatedly exposed to foliar pathogens, the composition of root exudates changes over time. These alterations result in the selection and accumulation of particular microbial communities within the soil, thereby strengthening the plant's defense against foliar pathogens (Vives-Peris et al., 2020; Yuan et al., 2018). In the case of wheat infected by *Puccinia striiformis*, similar secretions may occur. Several metabolites can promote the thriving of beneficial bacteria or fungi on the phyllosphere, which can be transported to the rhizosphere, either directly through the plant's tissues or via the air-soil interface (Feng et al., 2024; Philippot et al., 2013; Wang F. Y. et al., 2024). This phenomenon underscores the importance of the synergy between microorganisms in different ecological niches during plant disease resistance. The phyllosphere and rhizosphere microorganisms, influenced by the plant's hormonal and secretory responses to pathogen infection, work in harmony to combat diseases like wheat stripe rust (Luo et al., 2022; Yuan et al., 2018). When confronted with the attack of leaf pathogens, phyllosphere microorganisms function as the primary line of defense, sensing the pathogen and relaying signals to the rhizosphere (Fan et al., 2024; Li et al., 2023). In return, the rhizosphere microorganisms can provide additional support by enhancing nutrient uptake, generating antimicrobial substances, or regulating the plant's immune response (Liu et al., 2022; Xu et al., 2025). Notably, direct evidence linking phyllosphere signals to rhizosphere shifts remains limited. Future work will use LC-MS/MS to profile root exudates, qPCR to assess metabolite biosynthesis genes, and *in vitro* assays to test exudate effects on key taxa (Pang et al., 2021; Salem et al., 2022). This will clarify leaf-root crosstalk in microbial-mediated defense. Comprehending these intricate interactions is of vital importance for formulating sustainable approaches to manage plant diseases and enhance crop productivity.

## 5 Conclusion

**Figure 6:**
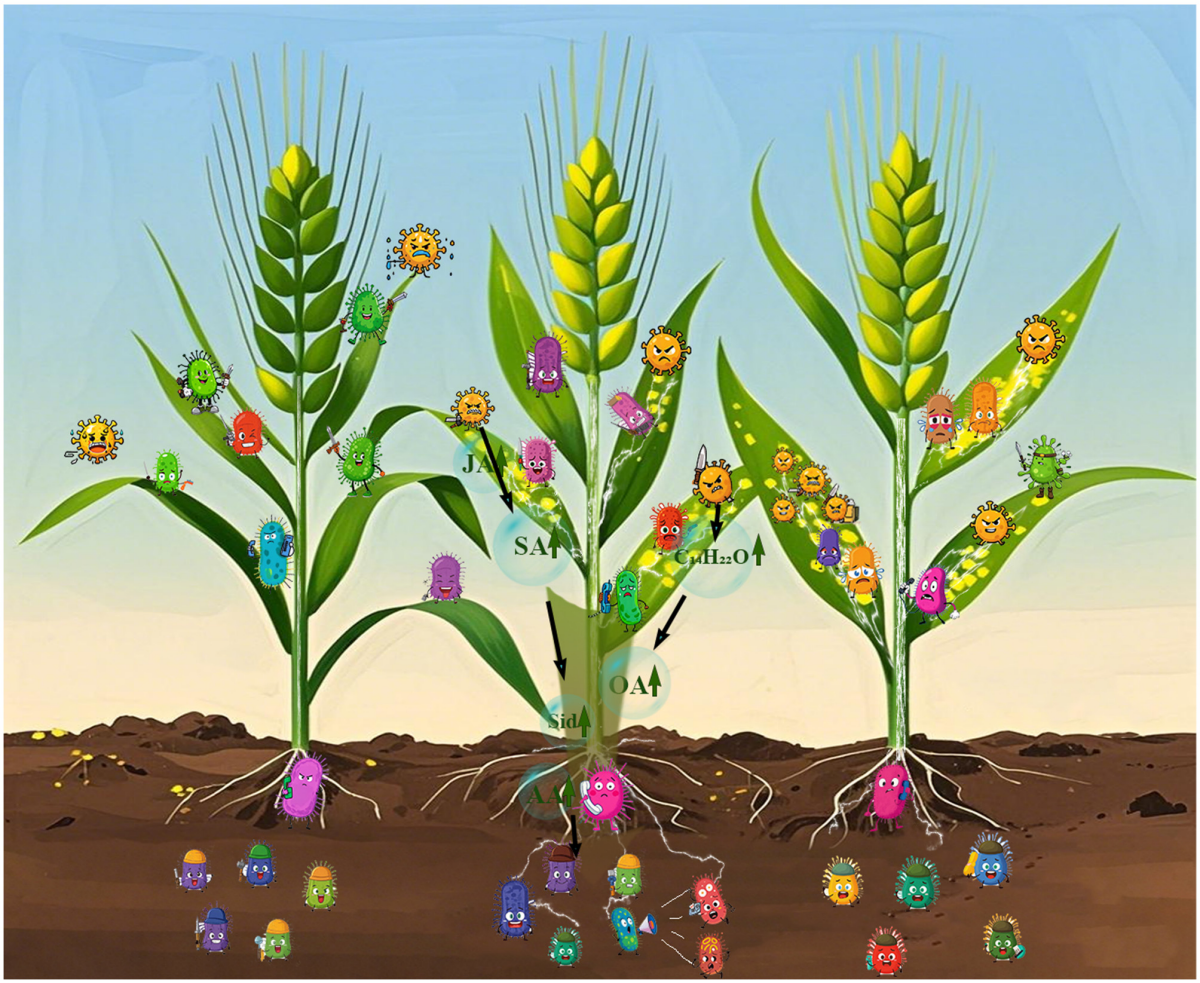
Different wheat varieties exhibit significant differences in their resistance to *Puccinia striiformis* infection. In the illustration, the three wheat plants demonstrate varying resistance levels. The **leftmost** wheat, with the highest resistance, has phyllosphere microbiota that can defeat the stripe rust fungus and alert its rhizosphere microbiome. The **middle** wheat, with lower resistance, shows phyllosphere microbiota in a stalemate with the pathogen—upon receiving the battle message, its rhizosphere microbiome “cries for help,” triggering the most pronounced changes. This alert is mediated by secondary metabolites produced in response to leaf damage, which translocate through the stem to the roots and are secreted into the soil surrounding the roots, thereby shaping the rhizosphere microbial community. For the **rightmost** wheat (with the lowest resistance), its phyllosphere microbiota are overwhelmed by the fungus; though the rhizosphere microbiome receives the “defeat” signal, it lacks effective countermeasures and stays in a passive state. 2,4-DTBP, 2,4-di-tert-butylphenol; Sid, siderophores; SA, salicylic acid; JA, jasmonic acid; OA, organic acids; AA, amino acids.

The research shows that wheat variety and *Puccinia striiformis* infection impact wheat's microbial communities in different niches. Variety dominates phyllosphere fungal structure, with fungal α–diversity increasing with resistance. In the rhizosphere, some microbial taxa abundances change among resistant varieties despite stable overall structure. The infection also alters community assembly randomness and network structure. Highly resistant varieties are better at strengthening microbial connections and resistance, but network stability weakens (Figure 6). The study deepens our understanding of the community responses, and future research can expand sampling and use multi-omics to reveal molecular mechanisms for better disease control.

## Data Availability

The datasets presented in this study can be found in online repositories. The names of the repository/repositories and accession number(s) can be found below: https://www.ncbi.nlm.nih.gov/, https://www.ncbi.nlm.nih.gov/sra/PRJNA1264767.
