# Supplementary material for: Distinct responses of wheat microbial communities in phyllosphere and rhizosphere to Puccinia striiformis infection
Source: Front Microbiol. 2025 Oct 7;16:1639152. doi: 10.3389/fmicb.2025.1639152 (PMC12537684; doi:10.3389/fmicb.2025.1639152)
Supplement: Supplementary file 1 [file Data_Sheet_1.docx]

**Supplementary Figures**

**
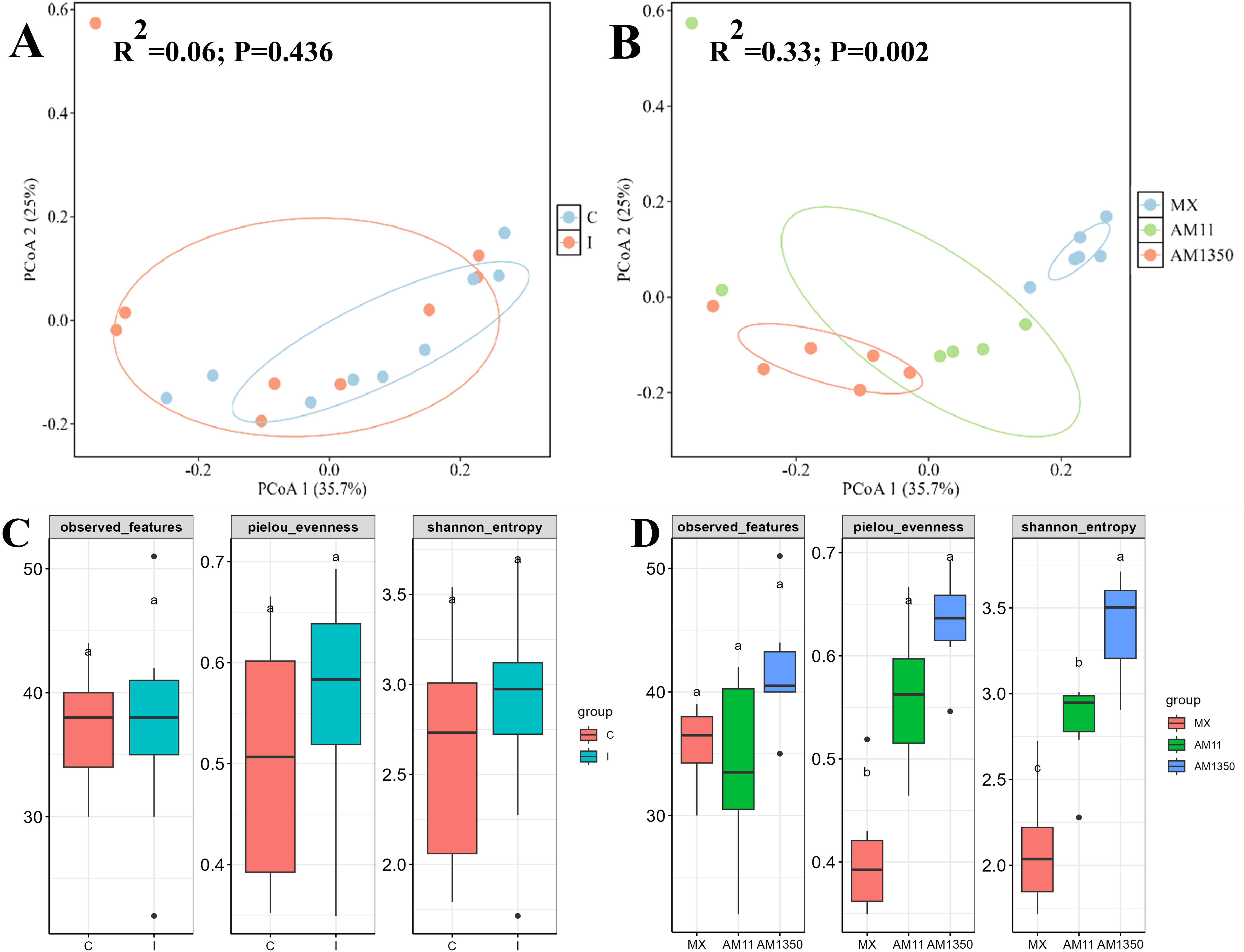
**

**Fig. S1.** Alpha diversity and Principal coordinate analysis (PCoA) in different treatments (A, C) and different varieties (B, D) of wheat phyllosphere fungi. For abbreviation: C: The control treatment; I: The inoculation treatment; MX: Samples of Mingxian wheat; AM11: Samples of Anmai 11 wheat; AM1350: Samples of Anmai 1350 wheat.


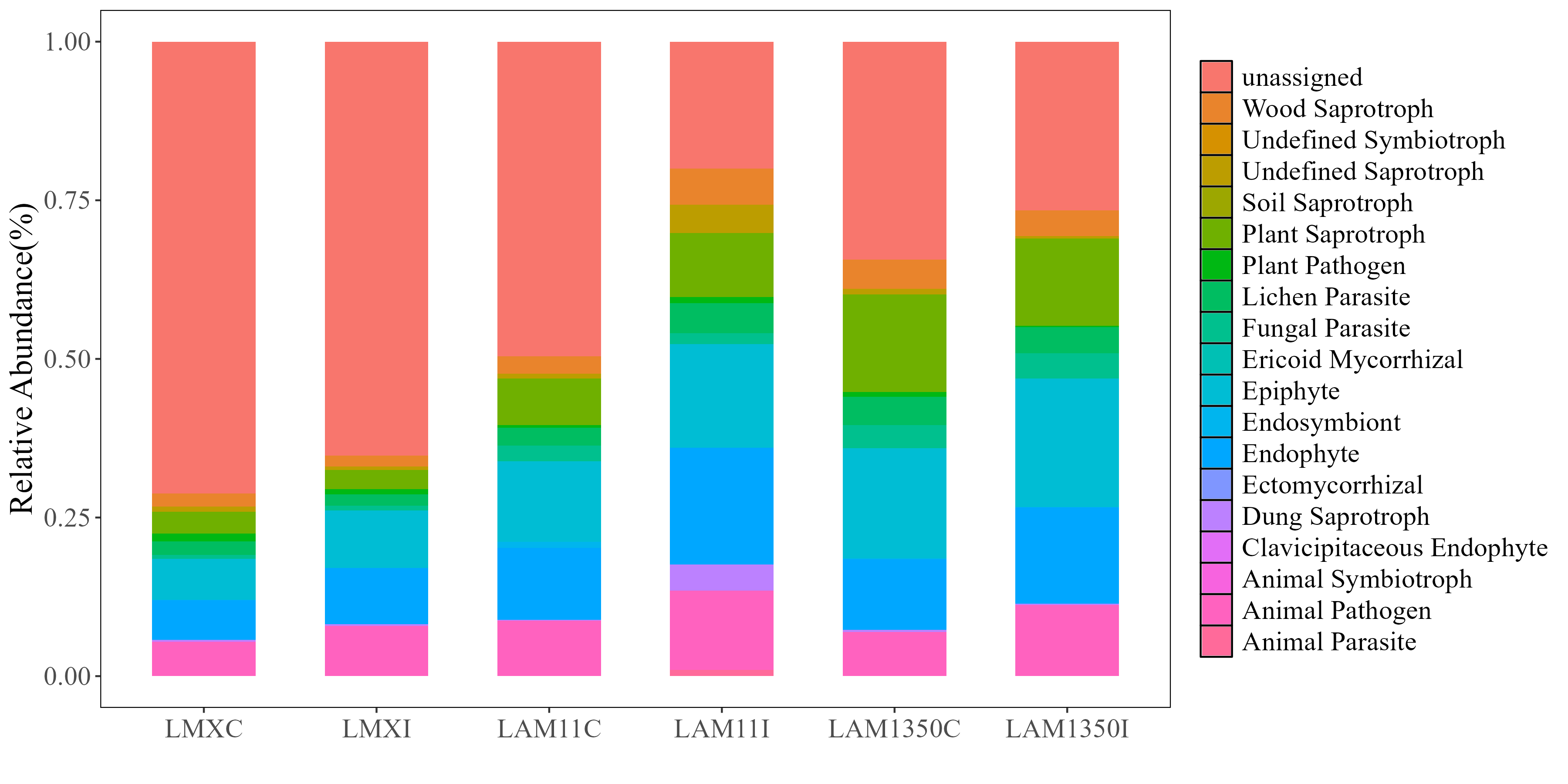


**Fig. S2.** Functional prediction of wheat phyllosphere fungal communities based on the FUNGuild database. For abbreviation: C: The control treatment; I: The inoculation treatment; MX: Samples of Mingxian wheat; AM11: Samples of Anmai 11 wheat; AM1350: Samples of Anmai 1350 wheat.

**
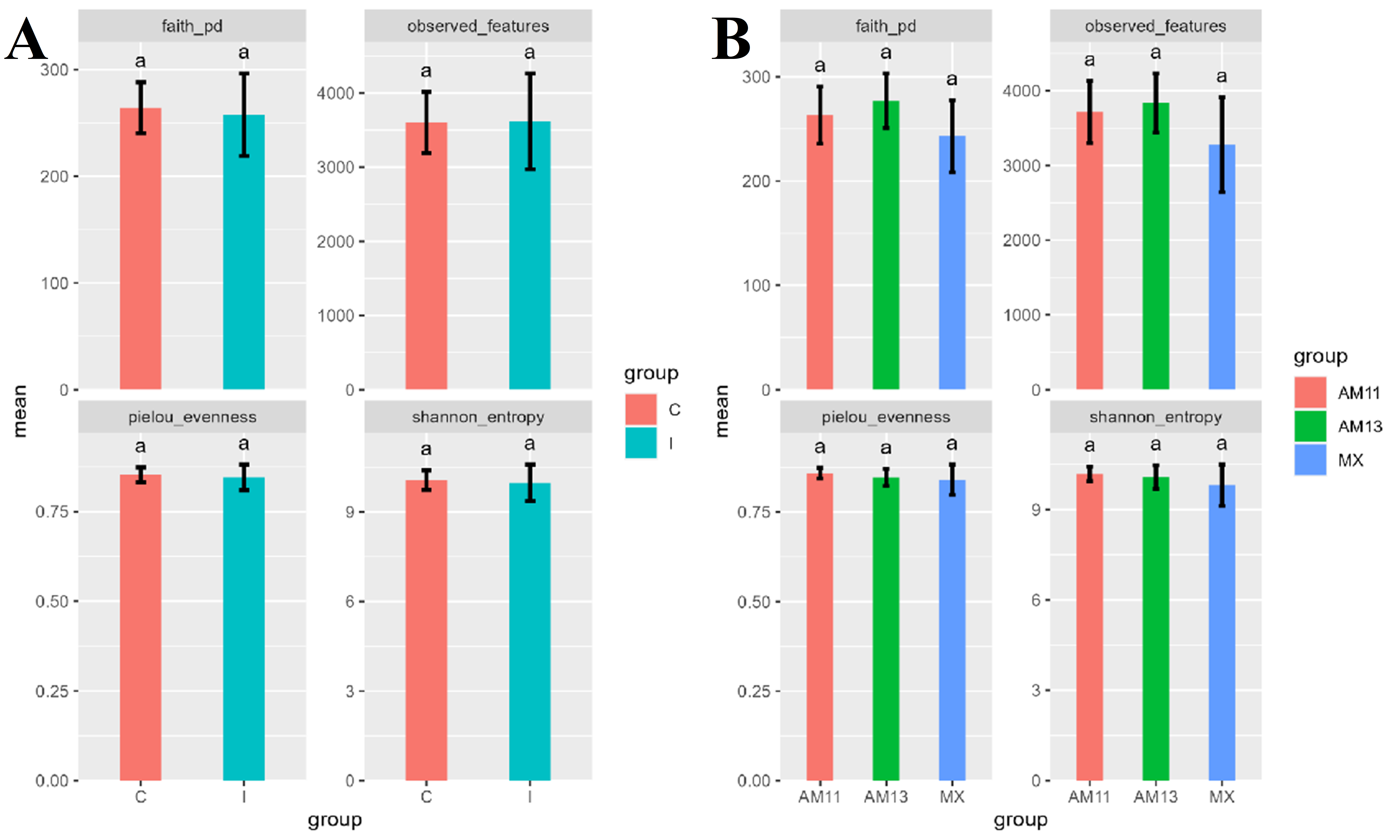
**

**Fig. S3.** Alpha diversity in different treatments (A) and different varieties (B) of wheat rhizosphere bacteria. For abbreviation: C: The control treatment; I: The inoculation treatment; MX: Samples of Mingxian wheat; AM11: Samples of Anmai 11 wheat; AM1350: Samples of Anmai 1350 wheat.

**
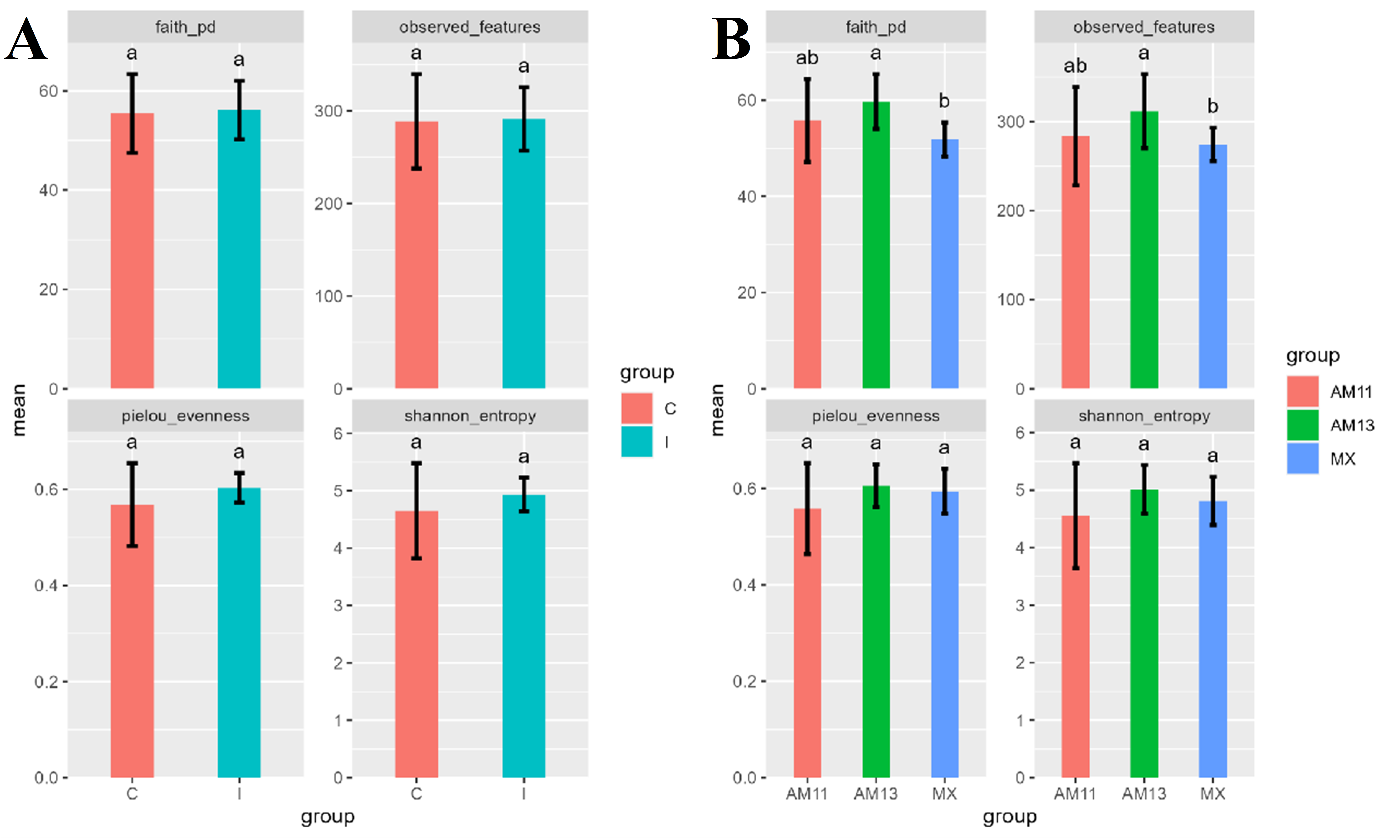
**

**Fig. S4.** Alpha diversity in different treatments (A) and different varieties (B) of wheat rhizosphere fungi. For abbreviation: C: The control treatment; I: The inoculation treatment; MX: Samples of Mingxian wheat; AM11: Samples of Anmai 11 wheat; AM1350: Samples of Anmai 1350 wheat.

**
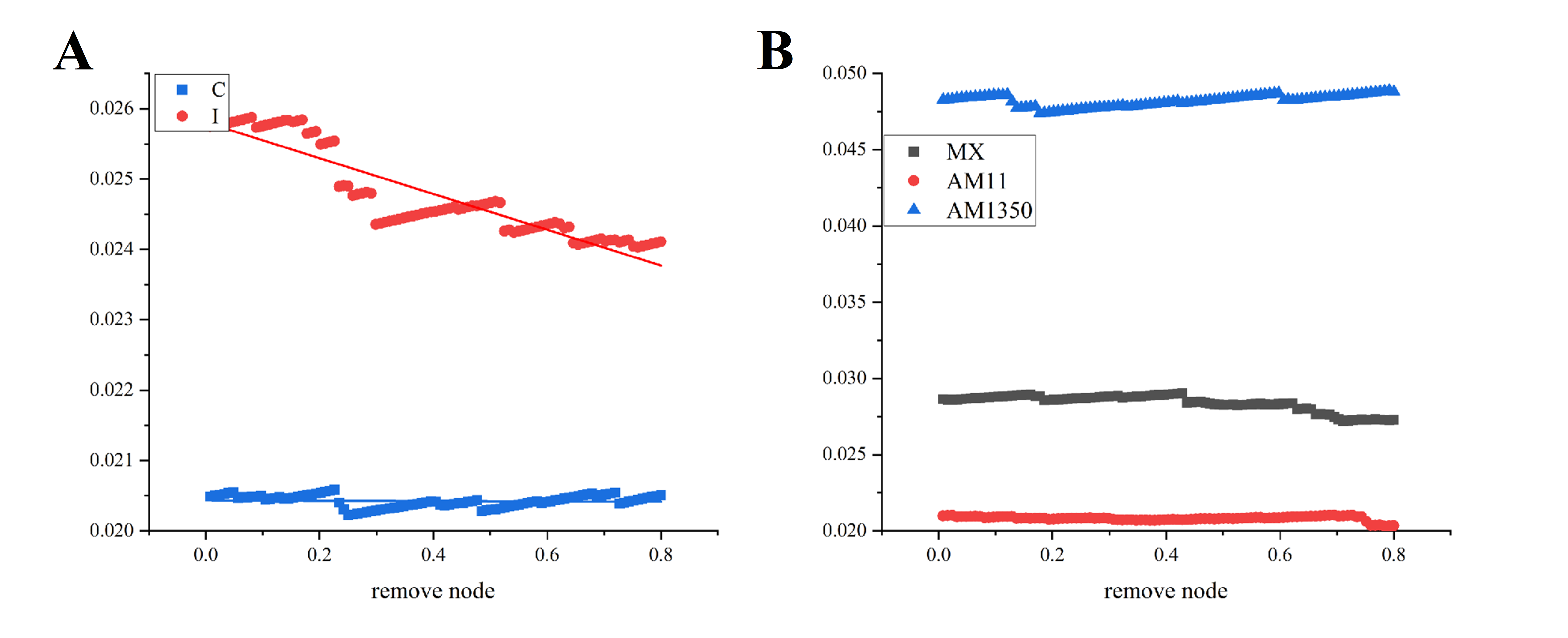
**

**Fig. S5.** Robustness analysis of co-occurrence networks in different treatments (A) and different varieties (B) of wheat rhizosphere. For abbreviation: C: The control treatment; I: The inoculation treatment; MX: Samples of Mingxian wheat; AM11: Samples of Anmai 11 wheat; AM1350: Samples of Anmai 1350 wheat.

**Supplementary Tables**

| **index** | **C** | **I** | **MX** | **AM11** | **AM1350** |
| --- | --- | --- | --- | --- | --- |
| **Nodes** | 1587 | 1639 | 1447 | 1584 | 1603 |
| **Edges** | 20145 | 21959 | 20726 | 22366 | 29984 |
| **Cluster** | 4 | 1 | 14 | 15 | 16 |
| **Edge density** | 0.02 | 0.02 | 0.02 | 0.02 | 0.02 |
| **Global clustering coefficient** | 0.36 | 0.38 | 0.52 | 0.51 | 0.55 |
| **Average clustering coefficient** | 0.36 | 0.38 | 0.53 | 0.53 | 0.54 |
| **Degree centralization** | 0.03 | 0.04 | 0.03 | 0.02 | 0.06 |
| **Betweenness centralization** | 0.00 | 0.01 | 0.01 | 0.01 | 0.02 |
| **Eigenvector centralization** | 0.93 | 0.94 | 0.92 | 0.89 | 0.91 |
| **Modularity** | 0.54 | 0.54 | 0.69 | 0.69 | 0.63 |
| **Complexity** | 12.69 | 13.40 | 14.32 | 14.12 | 18.70 |
| **Provincial Hub** | 14 | 12 | 7 | 7 | 5 |
| **Connector** | 109 | 120 | 12 | 23 | 50 |

**Table S1.** The topological characteristics of co-occurrence networks in different treatments and different varieties.
